# Dam Assisted Fluorescent Tagging of Chromatin Accessibility (DAFCA) for Optical Genome Mapping in Nanochannel Arrays

**DOI:** 10.1021/acsnano.2c12755

**Published:** 2023-05-08

**Authors:** Gil Nifker, Assaf Grunwald, Sapir Margalit, Zuzana Tulpova, Yael Michaeli, Hagai Har-Gil, Noy Maimon, Elad Roichman, Leonie Schütz, Elmar Weinhold, Yuval Ebenstein

**Affiliations:** † Department of Chemistry, Raymond and Beverly Sackler Faculty of Exact Sciences, Tel Aviv University, 6997801 Tel Aviv, Israel; ‡ Department of Biomedical Engineering, Faculty of Engineering, Tel Aviv University, 6997801 Tel Aviv, Israel; § Institute of Organic Chemistry, RWTH Aachen University, D-52056 Aachen, Germany

**Keywords:** chromatin accessibility, optical genome mapping, DNA labeling, methyltransferase labeling, nanochannels, single-molecule

## Abstract

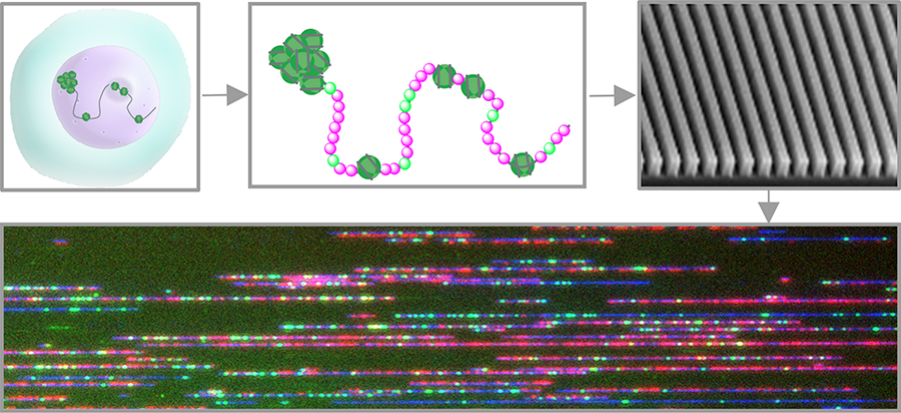

Proteins and enzymes in the cell nucleus require physical access to their DNA target sites in order to perform genomic tasks such as gene activation and transcription. Hence, chromatin accessibility is a central regulator of gene expression, and its genomic profile holds essential information on the cell type and state. We utilized the *E. coli* Dam methyltransferase in combination with a fluorescent cofactor analogue to generate fluorescent tags in accessible DNA regions within the cell nucleus. The accessible portions of the genome are then detected by single-molecule optical genome mapping in nanochannel arrays. This method allowed us to characterize long-range structural variations and their associated chromatin structure. We show the ability to create whole-genome, allele-specific chromatin accessibility maps composed of long DNA molecules extended in silicon nanochannels.

Chromatin is the packed form of eukaryotic genomes. It is a complex of macromolecules consisting of DNA, proteins, and RNA that enables genomic organization into microscopic nuclei. The basic structural unit of chromatin is the nucleosome, a complex consisting of DNA wrapped around assemblies of histone proteins.^1,2^ These complexes are further compacted into fibers of higher orders with increasing levels of physical density. Gene expression patterns in complex multicellular organisms are highly regulated by the physical and structural properties of chromatin.^3−7^ Thus, genomic chromatin patterns are characteristic of specific cell types and states. As one of the key players in the epigenetic control mechanism of gene expression, chromatin accessibility has become a main target of research for better understanding genomic processes in health and diseases.^6−10^

Common methods for assessing chromatin accessibility such as ATAC,^11,12^ Dnase,^13^ MNase,^14^ and FAIRE-seq^15^ are based on short-read next generation sequencing (NGS) and suffer from the inherent limitations of short-reads. Specifically, short-read sequencing is limited in its capacity to detect genomic structural variations (SVs) and copy number variations (CNVs) such as those in large repetitive elements.^16−21^ Such variations have a direct impact on genetic disorders and cancer, with established structural aberrations associated with a specific disease.^22−26^ The need to resolve large scale genetic structure has driven developments in cytogenetic technologies such as karyotype, fluorescence *in situ* hybridization (FISH), and arrays for comparative genomic hybridization (CGH).^25−27^ Optical genome mapping (OGM) is the most advanced cytogenetic technology, enabling full characterization of SVs and CNVs at high resolution.^28−31^ OGM is based on fluorescently labeling specific sequence motifs on high molecular weight DNA molecules extracted from cells or tissue. Single molecules are stretched in silicon nanochannels and imaged in their extended form to reveal an optical genetic pattern. The fluorescent patterns are then aligned to the human genome reference, identifying the chromosomal origin of the genomic fragment.^30−33^

Beyond genetic information, OGM offers additional genomic observables by use of additional labeling colors. Some examples include: DNA methylation and hydroxymethylation, DNA replication, and telomeres.^34−41^ Here, we present DAFCA (Dam Assisted Fluorescent tagging of Chromatin Accessibility), the latest addition to the epigenetic OGM toolbox (Figure 1). Previous work used non-CpG methyltransferases to methylate accessible genomic DNA within the cell nucleus. Specific DNA methylation is induced only in the accessible portions of the genome, which are then detected by methylation sensitive sequencing.

**Figure 1:**
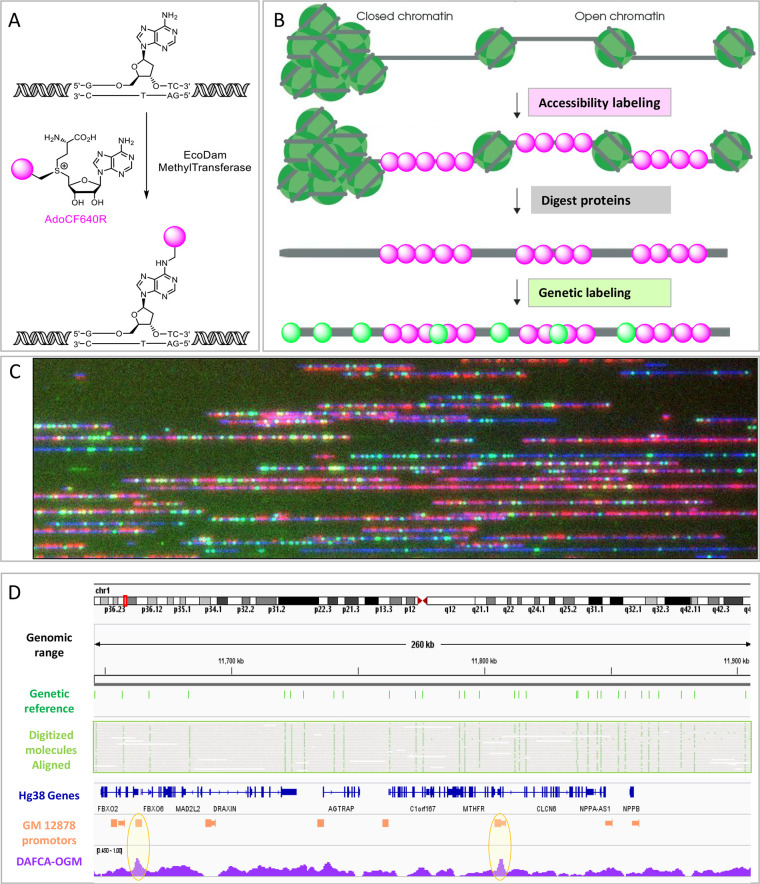
A. Molecular scheme for specific enzymatic DNA labeling using EcoDam MTase and a fluorescent cofactor. B. Schematic representation of the method concept from nuclei to dual labeled molecules. C. Representative field of view of dually labeled molecules. D. Genome browser view of a 260 Kbp region on chromosome 1, top to bottom; cytoband map; Hg38 genomic coordinates; predicted genetic sites; digitized representation of the mapped molecules to Hg38 according to their genetic labeling; Hg38 known genes locations; GM12878 cell-line known promoter locations; normalized DAFCA-OGM. Peaks of the DAFCA track that overlap with promoters are highlighted in orange circles, indicating highly accessible chromatin.

*E. coli* Dam (EcoDam) and other DNA methyltransferases (MTases) have already been shown to be suitable tools for reporting on chromatin accessibility by the detection of specific methylated sites with NGS and nanopore sequencing.^7,10,42−45^

We present a genome-wide chromatin profiling method based on optical mapping of individual, fluorescently labeled DNA molecules. EcoDam can transfer large chemical groups from cofactor analogues with extended methyl groups.^46^ When EcoDam is incubated with a fluorescent cofactor in permeabilized nuclei, only the accessible GATC sites within the chromatin are tagged and a fluorescent accessibility pattern is created (Figure 1A–D). We produced whole genome accessibility maps for the human lymphocyte cell line GM12878. These maps show distinct trends around genes and histone modifications in agreement with other NGS based methods (Figure 2).

**Figure 2:**
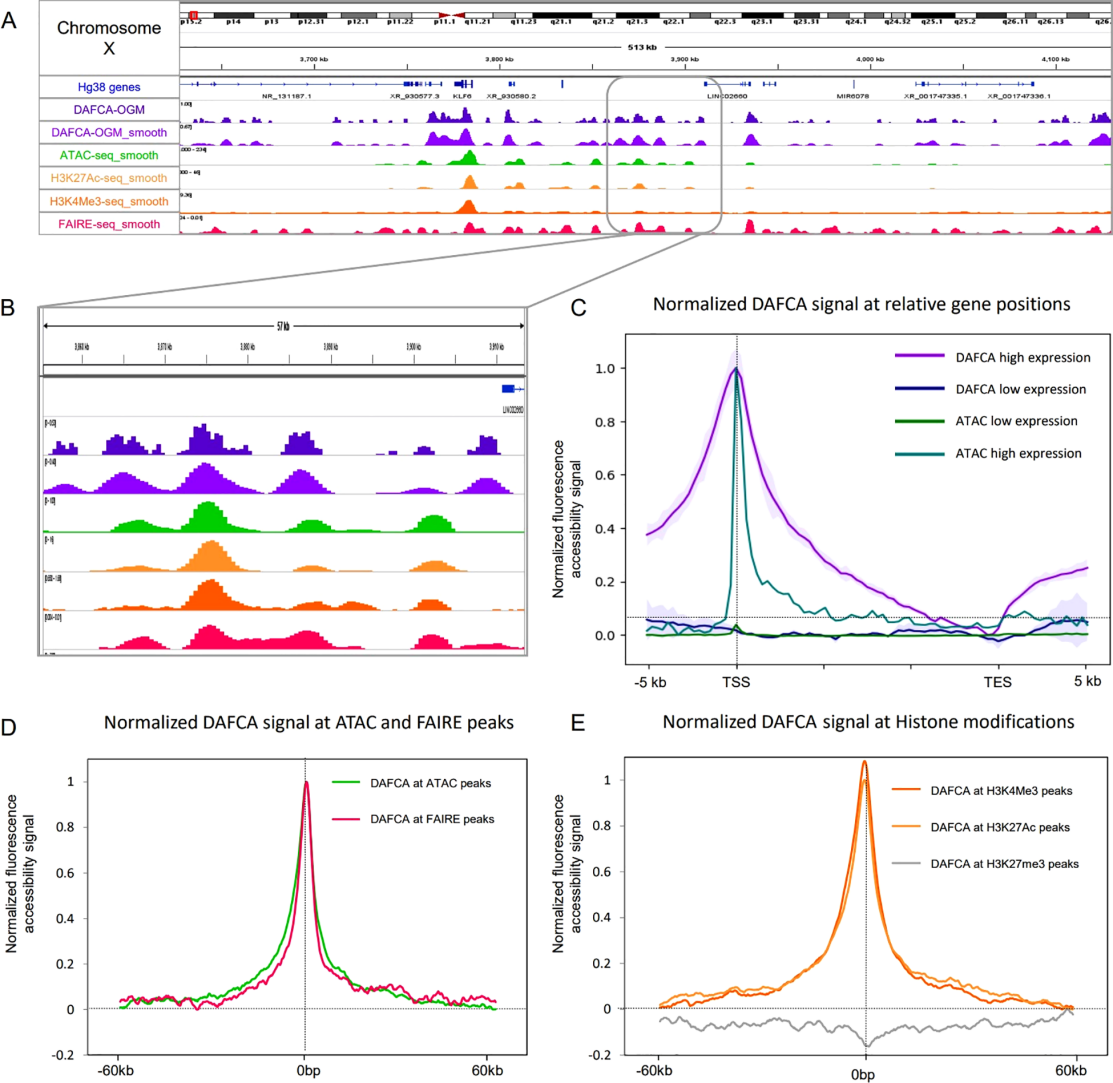
A and B. Genome browser view of a 513 Kbp region on chromosome X, top to bottom; cytoband map; normalized DAFCA- OGM; smoothed DAFCA- OGM; smoothed ATAC-seq; smoothed H3K27Ac; smoothed H3K4Me3; smoothed FAIRE-seq; All data tracks were downloaded from ENCODE and smoothed as described in the Methods section. C. Meta-analysis of the normalized Chromatin and ATAC signal along genes. Signals are plotted for two gene groups; 5000 most expressed genes and 5000 nonexpressed genes. Error bars for chromatin tracks display their STD. D. Meta-analysis of the normalized chromatin data as a function of distance from ATAC (green) and FAIRE (red) peaks. E. Meta-analysis of the normalized DAFCA signal as a function of distance from histone modifications peaks: H3K4Me3 (orange), H3K27Ac (yellow), and H3K27Me3 (gray).

Furthermore, using *de novo* assembly and SV analysis, we were able to profile genome-wide, allele specific chromatin accessibility for regions around SVs.^47^ We demonstrate the ability to assess the chromatin state of GAGE genes on chromosome X where the difference in accessibility determines the resistance to radiotherapy in ovarian cancer patients.^48^

## Results

### Method Validation

DAFCA generates a genome wide accessibility track where fluorescence intensity reports on the degree of chromatin accessibility. To validate the results obtained by DAFCA, we compared our genome-wide accessibility track to those obtained by existing chromatin accessibility methods such as ATAC-seq, FAIRE-seq, and accessibility histone marks (ChIP-seq).^12,15,49,50^ Two important factors had to be taken into account; the first is the optical diffraction limit, which limits the resolution of OGM to 500–1000 bp,^51^ and the second is the nonhomogeneous density of EcoDam labeling sites along the genome. To allow comparison, the sequencing data was binned and smoothed to match the resolution of the OGM experiment. EcoDam accessibility signal was normalized by the genome reference EcoDam site density track and an experimental “naked” control sample. The naked control was composed of genomic DNA stripped of histones and other DNA binding proteins such that all available EcoDam sites were fluorescently labeled, regardless of chromatin accessibility. Comparing the theoretical site density with the experimental fluorescence intensity in each 1Kbp bin allowed us to correct the signal generated from multiple labeling sites that are unresolved optically. Thus, after imaging and digitizing the labeled DNA molecules, the processed data is a fluorescence intensity signal along the genome. This track represents the chromatin open regions as a high signal and closed regions display a low signal. The full normalization and smoothing pipelines are detailed in the Methods section and the Supporting Information.

Figure 2A shows a region of 513 Kbp comparing the raw and smoothed DAFCA tracks to existing methods (ATAC-seq; ChIP-seq; and FAIRE-seq). A zoomed in region is shown in Figure 2B for more detailed inspection. It is clear that accessibility profiles generated by existing methods are well-represented by DAFCA; however, many additional signals are present in the DAFCA track. This is attributed to the relatively small size of the EcoDam enzyme, which allows it to access smaller open-chromatin regions, as will be discussed in the Conclusions section.^7,52^ Due to the resolution of OGM, smoothing was not required for downstream analysis and is shown here for presentation only.

To further establish the reliability of DAFCA, we performed a series of meta-analyses that examine the DAFCA signal around features characterized by the other methods. In Figure 2C, we display the distribution of chromatin and ATAC signals along genes. Two gene groups were defined, the first containing the 5000 highest expressed genes and the second containing 5000 nonexpressed genes. The highly expressed genes show a high accessibility signal, while nonexpressed genes are inaccessible, as previously reported.^6,53,54^ Despite the similar profile along genes between ATAC and DAFCA, the higher resolution of short-read sequencing is reflected in the narrower features. The error bars in the DAFCA plot display the variability in chromatin accessibility in the 5000 gene groups.

In Figure 2D, we show that accessibility levels recorded by DAFCA agree with those generated by both ATAC-seq and FAIRE-seq The maximum accessibility signal of DAFCA is maximal at ATAC-seq and FAIRE-seq peaks and rapidly declines with distance away from the accessible loci. In Figure 2E, we focus on histone modifications that are associated with the status of chromatin accessibility. We plot the DAFCA accessibility signal around peaks defined by ChIP-seq for specific chromatin associated histone modifications.^49^ For the two histone modifications that correlate with open chromatin (H3K4Me3 and H3K27Ac), we show a clear correlation between DAFCA and the regions of open chromatin, while no DAFCA signal is generated around histones associated with closed chromatin (H3K27Me3).

We note that all DAFCA plots display noisy data at highly condensed chromatin regions, this is due to the low labeling ability of EcoDam in these inaccessible regions, and thus lower fluorescence levels.

### Deciphering Allele Specific Chromatin Structure

The ability to decipher alleles based on their genetic profile allowed us to generate long-range allele-specific accessibility profiles overlaid on the genetic maps. The N50 length of our mapped molecules was 223 Kbp, enabling the generation of a highly contiguous *de novo* assembly.^47^ Using the Bionano Solve 3.6 algorithm, we generated a unique haplotype phased consensus map for the GM12878 cell line. We utilized this aspect of the OGM technology and created a specific reference for each allele (see the Methods section for more details).

**Figure 3:**
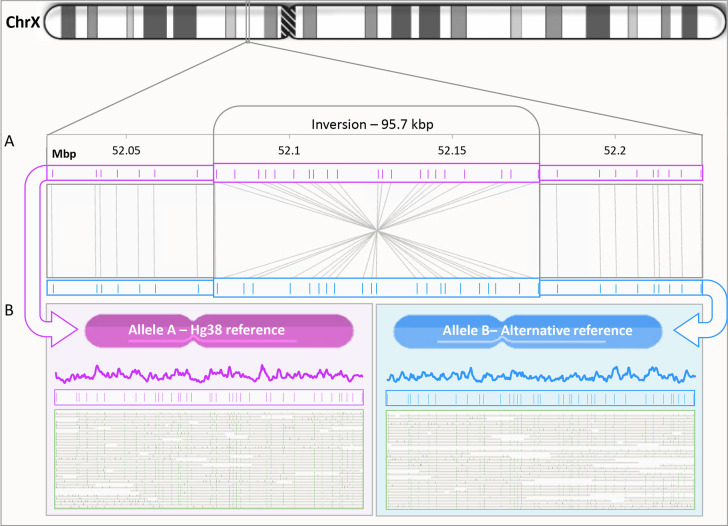
A. 95 Kbp inversion in chromosome X with inverted genetic pattern for each of the alleles; top pink is the Hg38 pattern, bottom blue track is the genetic pattern of the alternative allele map constructed by *de novo* assembly. B. For the same genetic region, we show for each of the alleles from top to bottom; DAFCA track; genetic labels pattern; and the aligned molecules from our data set used for the DAFCA track.

**Figure 4:**
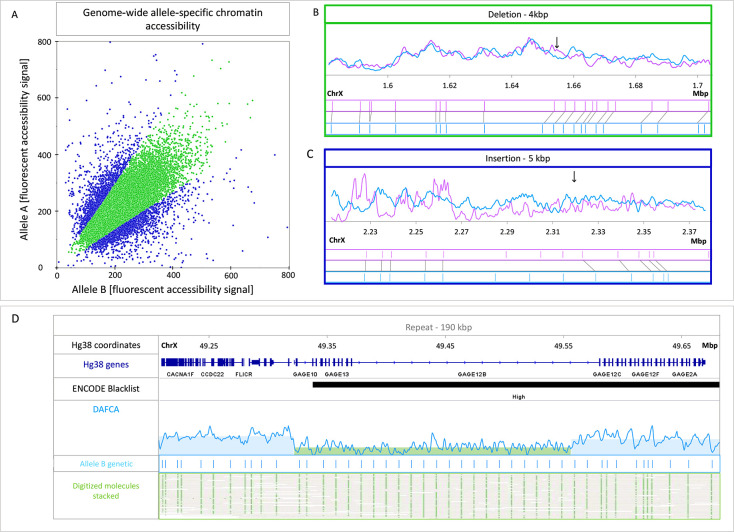
A. Scatter plot for the DAFCA bins around 350 detected heterozygous SV’s. Bins showing above 1.5-fold change in chromatin accessibility are presented in blue. B. Representative region for the green bins showing similar chromatin accessibility for both alleles around a 4 Kbp deletion in chromosome X. The two alleles are presented in light blue and pink with chromatin accessibility on top and the genetic pattern shown beneath. C. Representative region for the blue bins showing differential chromatin accessibility for both alleles around a 5 Kbp insertion. D. Genome browser view of a 90 Kbp repeat on chromosome X, top to bottom: Hg38 genomic coordinates, Hg38 known gene locations, ENCODE blacklist track (ambiguous region in the reference), normalized DAFCA signal, OGM genetic pattern, and digitized representation of the mapped molecules to the GM12878 *de novo* assembly.

For chromatin accessibility haplotype phasing, we aligned our chromatin maps to the different alleles using the green genetic labels, thus generating an accessibility map for each allele. We characterized allele specific accessibility maps for the whole genome and present data from chromosome X. The female identity of the GM12878 cell line is expected to display large variations in chromatin structure between the two copies of chromosome X.^55,56^ An example is shown in Figure 3, where a ∼100 Kbp inversion was used to distinguish between the two alleles and allowed us to determine the allele-specific chromatin pattern for both chromosome copies separately. Similarly, we were able to detect 350 unique heterozygous SVs. Figure 4A shows a scatterplot comparing the accessibility levels around these SVs on each of the two alleles. Data points on the plot represent a 1 Kbp bin in the vicinity of allele specific SVs. Altogether, we examined 35 Mbp of allele specific bins and found that 8.5% showed a distinct difference in the accessibility signals between the two alleles. Bins presenting over 1.5-fold change were arbitrarily defined as highly differential regions. while the majority of bins displayed similar accessibility profiles. When analyzing all 2959 highly differential regions, 1269 overlapped with genes, 170 with promoters, and 459 with known enhancers (see Table S1 in the Supporting Information). Interestingly, when compared to the overall distribution of genetic features, we see a clear enrichment of differential accessibility at promoter regions (48% higher abundance compared to genes and enhancers, Table S2 in the Supporting Information). Clinically relevant thresholding of regional chromatin accessibility may be established upon accumulation of statistically significant data.

Parts B and C of Figure 4 show two examples of allele specific SVs and their associated accessibility profiles. These two examples represent the two types of loci exhibited in the scatter plot in green and blue. The first (Figure 4B) shows two genetically variable alleles with similar accessibility profiles, while the second displays differential accessibility between the alleles (Figure 4C). A third example emphasizes the ability of DAFCA to characterize the chromatin structure in highly repetitive regions, a challenging task for short-read sequencing-based technologies.^16^ Specifically, Figure 4D depicts the genetic structure of the GAGE 12 region, identifying the exact copy number.^47^ Furthermore, DAFCA provides the accessibility profile along this repeat array, recognizing a distinct drop in chromatin accessibility along GAGE12.

## Conclusions

This work establishes a chromatin accessibility assessment method that relies on optical investigation of individual DNA molecules confined in silicon nanochannels. Utilizing a chemo-enzymatic protocol, our assay integrates into the commercially available OGM technology from Bionano Genomics Inc. The combination of OGM long reads with chromatin accessibility labeling gives access to complex genomic structural variants and their unique chromatin status. Additionally, allele-specific chromatin structure is resolved based on detecting heterozygous SVs with OGM.

Several methods for assessing chromatin accessibility have been developed in the last 15 years. The different methods usually agree for large portions of the genome but differ in the fine details they provide, displaying differences in resolution and peak density as shown in Figure 2A.^12,15,49^ Greenleaf and co. have suggested that these differences arise from the physical size of the chromatin sampling enzyme used by each method.^7^ Of particular relevance is the higher peak density of DAFCA compared to ATAC, as seen in Figure 2. We attribute the difference mainly to the small size of Dam (36 kDa),^57^ which can access smaller gaps between compact chromatin regions compared to the larger Tn5 Transposase (53 kDa)^58^ used for ATAC.

Recent years have seen a boost in the relevance of chromatin accessibility data to the clinic.^8,9,59^ An interesting recent finding links the density of chromatin to the expression of GAGE 12. High expression of GAGE 12 results in relaxing of the chromatin and promotes resistance to radiation therapy in cervical cancer.^48,60^ Due to the relation between chromatin density and gene expression (Figure 2), it is possible to evaluate the expression level of a certain gene *via* analysis of its chromatin state. Nevertheless, most GAGE genes are located within the ENCODE blacklist,^61^ a list of regions in the human genome that have anomalous, unstructured, or high variation in NGS studies. Such regions should be removed during data analysis as they provide an unreliable snapshot of the genome. This fact makes it difficult to accurately resolve the chromatin state of GAGE 12 by NGS-based techniques due to its repetitive nature.^62^ As proof of concept, we show the ability of DAFCA to recognize a distinct drop in chromatin accessibility along GAGE12 in the GM12878 cell line. This region of condensed chromatin implies low expression of the gene, which in turn would indicate less resistance to radiation therapy. Such information requires multiple sequencing assays when using NGS approaches and showcases the potential advantage of DAFCA in clinical settings.

To conclude, we present an approach for simultaneous optical genomic mapping of genetic structure and chromatin accessibility. DAFCA integrates into commercially available OGM at almost no extra cost and provides important complementary data. Beyond genome-wide chromatin accessibility profiling, DAFCA was able to map molecules and profile their accessibility pattern in repetitive regions and heterozygous SVs, which are extremely challenging for NGS-based methods.

## Methods

### Cell Culture

GM12878 cells were purchased from the Corriel Institute. Cells were grown in clonal populations under the recommended conditions: medium, RPMI 1640, 2 mM l-glutamine, and 15% fetal bovine serum; culture conditions, T25 tissue culture flask with 10–20 mL medium upright position at37 °C under 5% carbon dioxide. Then, 22 × 10^6^ cells (2 × 30 mL of 37 × 10^4^ cells per mL) were centrifuged for 5 min at 1000*g* in two 50 mL conical tubes, followed by two 50 mL washes in cold PBS (−Ca, −Mg) for 5 min at 1000 RCF for both falcons. Final pellets were each suspended in 500 μL of cold RSB by gentle flicking, transferred to two 15 mL conical tubes, and kept on ice. Each of the 50 mL tubes were washed one more time with 500 μL of cold RSB that was added to the corresponding 15 mL conical tube to a final volume of 1 mL of cells in RSB.

### Nuclei Permeabilization and Isolation

Each of the cell-containing falcons was gently lysed with cold RSB + 0.1% NP40 (total of 14 mL). The tubes were inverted 5–10 times and centrifuged at 1000*g* for 15 min at 4 °C to pellet nuclei. The supernatant was removed, and the tube was kept upside down to dry for a few minutes. Cell pellets were resuspended in 60 μL of EcoDam buffer (150 mM Tris-HCl, 10 mM EDTA, 2 mM DTT, pH 8.0); at this point, both pellets were untied to give 120 μL that was calculated to give ∼20 × 10^6^ nuclei per mL of concentration. Half of the volume was directly used for the chromatin accessibility labeling, and the other half served for the naked control sample that continued straight to high-molecular-weight DNA extraction.

### Chromatin Accessibility Labeling

Synthesis of the AdoYnCF640R labeling reagent was previously reported.^63^ The expression protocol for EcoDam is presented in the Supporting Information. This labeling step was performed on isolated and permeabilized nuclei. Labeling was applied in three separate reactions to maintain individual reaction volumes of 20 μL each: 17 μL (2 μg DNA) of nuclei in EcoDam buffer with 0.25 μL of EcoDam (51 μM, 1.6 μg/μL) and fluorescent cofactor (AdoYnCF640R) at a final concentration of 50 μM in a final volume of 20 μL (adjusted to the final volume with EcoDam buffer). Samples were incubated for 1 h at 37 °C and combined to a final volume of 60 μL.

### Naked Control Labeling

Labeling of this sample was performed under the same conditions as the chromatin accessibility sample, directly after the high molecular weight DNA extraction step. After labeling of the naked control DNA, 5 μL of proteinase K (Qiagen) was added and the reactions were incubated at 50 °C for 30 min and then heated to 80 °C for 20 min for the red cofactor (AdoYnCF640R) deactivation.^35^ The three samples were combined, and excess fluorophore cleanup was done by two subsequent drop dialysis steps using 0.1 μm dialysis membrane (Millipore) floating on 15 mL of TE in a 6 cm Petri dish. The mixture was mixed 5 times with a commercial wide-bore tip and added as a single drop on the center of the dialysis membrane. The Petri dish was covered and protected from light, while the sample was dialyzed for 1 h at room temperature; then, the drop was transferred to a different location on the membrane for another 0.5 h. The collected 40 μL DNA mixture was gently pipetted and incubated at RT overnight to achieve homogeneity of the high molecular weight DNA. The following day, EcoDam labeled naked DNA concentration was determined as 142 ng/μL by the Qubit BR dsDNA assay (Thermo Fisher).

### High Molecular Weight DNA Extraction

High molecule weight (HMW) DNA was extracted from 60 μL of the chromatin accessibility labeling reaction using a Bionano Prep Blood and Cell Culture DNA Isolation kit according to the supplied protocol (Bionano Genomics). DNA concentration was determined to be 107 ng/μL for the chromatin sample by the Qubit BR dsDNA assay.

For the naked control sample, this step was done directly after nuclei permeabilization and isolation; DNA concentration was 70.3 ng/μL.

### Genetic Barcoding and Staining for Optical Genome Mapping

The chromatin and naked samples were labeled by Direct Label and Stain (DLS) chemistry (DLE-1 enzyme, CTTAAG motif), creating a genetic barcode according to a protocol by Bionano Genomics (https://bionanogenomics.com/support-page/dna-labeling-kit-dls/).

### Imaging

Labeled DNA molecules were loaded into commercial nanochannel arrays where they were confined and extended (Saphyr chip G1.2). Nanochannels were automatically imaged to acquire 728 Gbp of individual DNA molecules longer than 150 Kbp for the chromatin sample and 350 Gbp for the naked control sample. Three layers of data were collected from each field of view, DNA backbone in blue, genetic (DLE-1) barcode in green, and chromatin accessibility (EcoDam) profile in red, as seen in Figure 1C (Saphyr, Bionano Genomics).

### Optical Chromatin Accessibility Mapping Analysis

For analysis of the high-throughput nanochannel array data, raw images were processed, and DNA molecules were detected and digitized by custom image-processing and analysis software (Saphyr Molecule Detect, Bionano Genomics). Briefly, a set of coordinates along the molecules was assigned to genetic (DLE-1) labels. The accessibility labeling (EcoDam) was digitized as a continuous intensity profile along the DNA molecules.

Using Bionano Access (v1.6.1) and Bionano Solve (v3.6.1), the digitized molecules were aligned to the reference, according to the coordinates of their genetic pattern. Molecules spanning over 150 Kbp were aligned to the *in silico* human genome reference GRCh38.p13 (Hg38 reference), based on DLE-1 recognition sites (*hg38_DLE1_0kb_0labels.cmap*), with default parameters matching the following combination of arguments: haplotype, human, DLE-1, Saphyr. Only molecules with an alignment confidence equal to or higher than 17 (*P* ≤ 10^–17^) and that at least 60% of their length was aligned to the reference were used for downstream analysis. Alignment outputs were converted to global fluorescent profiles (bedgraph files).^31,35^ The full pipeline and parameters used can be found in the Supporting Information.

### Data Normalization

Due to the nonhomogeneous distribution of EcoDam sites in the genome, a normalization step based on this distribution was necessary. For example, two open chromatin regions may have a different number of EcoDam sites, resulting in different fluorescent signals. This may be corrected by comparing the control naked sample (where all EcoDam sites are accessible) to the theoretical EcoDam site distribution in the genome reference. By correlating the number of theoretical EcoDam sites per bin with the fluorescence intensity for that bin, we get a conversion factor for normalizing the fluorescence data. Bionano Genomics Access was used to generate a predicted genomic track of EcoDam binding site motifs (GATC) on the Hg38 reference. We used the python SciPy smooth package “SciPy 1.0” with parameters set to a window size of 4000 bp, STD value of 2000 bp, and overlapping of 500 bp.^64^ The smoothing parameters were chosen to provide the best fit between the experimental and theoretical DAM site distribution. These parameters were applied only on the theoretical track, while the raw data was used as-is for further analyses. Further details on the establishment of these parameters are given in the Supporting Information. We used Bedtools *makewindow* (v2.26.0) followed by Bedtools *map* (v2.26.0) to bin all data sets into 500 bp nonoverlapping genomic windows of hg38.^65^ Using Python data sciences tools,^66^ we found that the naked and predicted data fit a linear regression model with the formula of: signal = 58.93 × (sites) + 216.18; this formula connects the number of predicted EcoDam sites with the recorded fluorescence intensity for each bin (Figure S1A,B). We applied the reverse equation in order to normalize the chromatin accessibility track: , resulting in a normalized track representing the true chromatin state along the genome (Figure S1C) regardless of the local site distribution.

### Comparing DAFCA Tracks to Existing NGS Methods

Sequencing-based accessibility profiling data sets for GM12878 using ATAC-seq, FAIRE-seq, and ChIP-seq (H3K4me3, H3K27ac, H3K27me3) were downloaded from the ENCODE portal (https://www.encodeproject.org/)^67,68^ with the following identifiers: alignment tracks, ENCFF240YRV, ENCFF000THZ, ENCFF287HAO, ENCFF469WVA, and ENCFF486WAD. Data was smoothed as described above. The following peak tracks were used for meta-analysis: ENCFF118WLS, ENCFF000THW, ENCSR057BWO, ENCFF835NLI, and ENCFF485JZA.

### Meta Analysis

Transcription start and end sites (TSS and TES) of protein-coding genes were defined according to GENCODE annotation (v34);^69^ gene bodies were defined as spanning from the transcription start site (TSS) to the transcription end site (TES).

RNA-seq for GM12878 was download from the ENCODE project (ENCFF853VUK).^67,68^ Protein-coding genes were divided into two groups based on their average normalized TPM score. The top 25% with the highest score of transcripts per million (TPM) values was 27902: 23 (4909 genes) expressed genes and unexpressed genes with TPM value of 0 (5030 genes). Mean DAFCA/ATAC (ENCFF240YRV) signals along genes were calculated using DeepTools *computeMatrix* (v3.4.1)^70^ in scale-regions mode, where each gene was scaled to 15 Kbp and divided into 300 bp bins.

### Optical Chromatin Accessibility Signals around NGS-Based Accessibility Peaks

Mean chromatin signals around accessibility peaks generated by three other methods were calculated. The calculation was performed with default DeepTools *computeMatrix* (v3.4.1)^70^ parameters, and the length was set to 300bp.

### *De Novo* Assembly and Haplotyping

Optical mapping data for the GM12878 cell-line samples was merged to a single data set using Bionano Access (v1.6.1) and Bionano Solve (v3.6.1) and used for *de novo* assembly of the GM12878 genome. The parameters used were ‘haplotype with extend and split’ and “cut CMPR”. The human genome GRCh38.p13 (*hg38_DLE1_0kb_0labels.cmap*) was used as the reference. The analytical pipeline outputs contiguous maps (contigs) of the sample’s specific genomic structure. It is haplotype sensitive and displays different contig maps for different alleles. These maps are ultimately compared to the Hg38 reference in order to call for SV and CNV events at regions where the genetic pattern differs from the reference.

### Unique SVs and Their Accessibility Profile

We selected 134 contiguous maps larger than 300 Kbp that displayed at least one allele-specific SV event and constructed separate genetic maps for the two alleles. Using the list of detected SVs, we identified 350 heterozygous SVs present only in our *de novo* constructed genetic map. We then aligned the chromatin accessibility patterns recorded in 100 Kbp regions around the SV to generate the allele specific chromatin maps. The signal intensity in these maps was summed in 1 Kbp bins. The pairwise ratio between corresponding bins of each allele-specific accessibility map was calculated and plotted as a scatter plot using python scatter plot function from the *seaborn library*.^71^ Bins presenting over 1.5-fold change were defined as highly differential and are shown in blue in Figure 4C.

## Data Availability

https://zenodo.org/record/7701717#.ZAXatXZBwdU and https://github.com/ebensteinLab/DaFCA

## Supporting Information Available

Discussions of chromatin accessibility signals along aggregated genetic features, expression of *E. coli* Dam (EcoDam), conversion of OGM data to global chromatin accessibility maps, data smoothing, and correlation between DAFCA replicates and figures of CC values for different window sizes and scatter plots demonstrating the correlation between replicates (PDF)
