# Supplementary material for: Dam Assisted Fluorescent Tagging of Chromatin Accessibility (DAFCA) for Optical Genome Mapping in Nanochannel Arrays
Source: ACS Nano. 2023 May 8;17(10):9178–87. doi: 10.1021/acsnano.2c12755 (PMC10210529; doi:10.1021/acsnano.2c12755)
Supplement: Supplementary file 1 — nn2c12755_si_001.pdf [file nn2c12755_si_001.pdf]

Supporting Information

Dam Assisted Fluorescent tagging of Chromatin Accessibility (DAFCA) for optical genome mapping in nano-channel arrays.

Gil Nifker[1], Assaf Grunwald[1], Sapir Margalit[1], Zuzana Tulpova [1], Yael Michaeli[1], Hagai Har-Gil[1], Noy Maimon[1], Elad Roichman[1], Leonie Schütz[3], Elmar Weinhold[3]* and Yuval Ebenstein[1,2]*.

[1] Department of Chemistry, Raymond and Beverly Sackler Faculty of Exact Sciences, Tel Aviv University, 6997801 Tel Aviv, Israel.
[2] Department of Biomedical Engineering, Faculty of Engineering, Tel Aviv University, 6997801 Tel Aviv, Israel.

[3] Institute of Organic Chemistry, RWTH Aachen University, D-52056 Aachen, Germany.

*To whom correspondence should be addressed Email: uv@tauex.tau.ac.il, elmar.weinhold@oc.rwth-aachen.de.

1. Chromatin accessibility signals along aggregated genetic features

Table S1

Number of bins overlapping with genetic features	All SV bins	SV in Gene bodies	SV in promotors	SV in enhancers	SV in non-coding regions
Total	35053	16769	1368	6744	10172
Highly differential regions	2959	1269	170	459	1061

Table S2

% Bins overlapping with genetic features	Gene bodies	Promotors	Enhancers	Non-coding
Total	48%	4%	19%	39%
Highly differential regions	43%	6%	16%	35%

Gene bodies were defined as spanning from the transcription start site (TSS) to the transcription end site (TES) annotated by GENCODE v34.[1] Promoters were defined as ranging from 1000 bp upstream to 500 bp downstream from the GENCODE TSS. General predicted enhancers were mapped to gene targets by JEME and adapted from Cao et al. [2] Genomic coordinates of enhancers were converted from the human genome build hg19 to hg38 using UCSC liftOver.[3] Enhancers overlapping ambiguous genomic regions were discarded, as well as pairs of enhancers and gene targets that are overlapping or in close proximity (up to 5 kbp).[4] The number of bins overlapping each of the genetic features was calculated using Bedtools *intersect* (v2.26.0).

2. Expression of E.coli Dam (EcoDam)

The EcoDam gene from pDOX1 vector was sub-cloned as a R.NdeI-R.SalI fragment into the multiple cloning site of pET-22b(+) to obtain a pET-22b(+)/EcoDam expression vector.[5] Dam-deficient T7 expression strain ER2948, from Dr. Elisabeth Raleigh's strain collection at New England Biolabs, was transformed with the pET-22b(+)/EcoDam plasmid and grown on LB plates containing ampicillin (100 mg/L) at 37°C overnight. The next morning LB medium (3 mL; 10 g/L tryptone, 5 g/L yeast extract, 10 g/L NaCl, pH 7.0) containing ampicillin (100 mg/L) was inoculated with a colony of ER2948/pET-22b(+)/EcoDam cells and incubated by shaking at 250 rpm and 37°C. In the evening 0.5 mL of this cell culture were transferred into LB medium (300 mL) containing ampicillin (100 mg/L) and incubation was continued with shaking at 37°C overnight. Five flasks with LB medium (2 L each) containing ampicillin (100 mg/L) were inoculated with this overnight starter culture (20 mL for each flask) the next morning and cells were grown at 37°C until OD_{600} reached about 0.6. Protein expression was induced by adding a sterile IPTG solution (0.1 mM final concentration) and the cell cultures were incubated by shaking at 20°C for 3.5 h. Cells were harvested by centrifugation at 3,500 rpm and 4°C for 15-20 min, the supernatant was carefully discarded and the pellet stored at -20°C.

Protein purification: The cell pellet was resuspended in ice cold lysis buffer (5 mL/g wet cells; 500 mM NaCl, 100 mM Tris-HCl, 10 mM EDTA, 5 mM DTT, 2 mM PMSF (prior dissolved in ethanol), 10% glycerol, pH 7.6) and sonicated (output control 8, duty cycle 60%) on ice for 3 x 1 min with cooling periods in between. Crude cell lysate was centrifuged at 16,000–17,000 rpm and 4°C for 1 h to remove cell debris and the cleared lysate was supplemented with four volumes of buffer A (50 mM K_2HPO_4/KH_2PO_4, 1 mM EDTA, 2 mM DTT, 5% glycerol, pH 7.6). The diluted lysate was loaded onto a cation-exchange column (Poros HS 50, 20 mL) which was equilibrated before with buffer A containing KCl (0.1 M). Proteins were eluted with a linear KCl gradient (0.1 to 1 M) in buffer A and fractions analyzed by SDS-PAGE. Fractions containing EcoDam were combined and concentrated using centrifugal concentrators with 10 KDa cut off at 4°C. The concentrated protein solution (about 5 mL) was loaded onto a gel filtration column (Superdex 75, 320 mL) which was equilibrated before with gel filtration buffer (100 mM KCl, 100 mM Tris-HCl, 20 mM EDTA, 2 mM DTT, 5% glycerol, pH 7.5). Proteins were eluted with gel filtration buffer and fractions analyzed by SDS-PAGE. Fractions contained EcoDam were combined and loaded again onto the cation-exchange column (Poros HS 50, 20 mL) but this time equilibrated with cofactor releasing buffer (300 mM KCl, 6.7 mM MES, 6.7 mM NaOAc, 6.7 mM HEPES, 0.2 mM DTT, 10% glycerol, pH 6.0). The column was extensively washed with cofactor releasing buffer (9 L) and treated with buffer B (100 mM Tris-HCl, 20 mM EDTA, 2 mM DTT, 5% glycerol, pH 7.5) containing KCl (0.1 M). Proteins were eluted with a linear KCl gradient (0.1 to 1 M) in buffer B and fractions analyzed by SDS-PAGE. EcoDam containing fractions were combined, concentrated by ultrafiltration (10 KDa cut off), supplemented with glycerol (50% final concentration) and stored at -20°C. EcoDam concentration was determined by

UV-Vis spectroscopy in buffer (200 mM KCl, 100 mM Tris-HCl, 10 mM EDTA, 2 mM DTT, 0.1% n-dodecyl β-D-maltoside, pH 7.5) using an extinction coefficient at 280 nm of 37,400 L mol^{-1} cm^{-1} and a molecular weight of 32,000 g/mol. Typically, 34 mg of purified EcoDam were obtained from 10 L cell culture.

3. Conversion of OGM data to global chromatin accessibility maps

All scripts mentioned in this work are available from: https://github.com/ebensteinLab/DaFCA, https://github.com/ebensteinLab/EcoDAM

The data output from the Bionano Genomics "Saphyr Molecule Detect" is provided in proprietary CIP and BNX formats. The CIP file contains the indexing information necessary for retrieving the continuous intensity profile for a specific molecule in a BNX file. The file is an ASCII text, tab delimited file. Which is not compatible with commonly used bioinformatic tools. Therefore, the intensity profiles of the red channel (EcoDam accessibility labeling) for the filtered molecules were converted by a series of custom python scripts:

The first is *readCIP.py* that converts the data into a numerical **profileTrace.txt** file containing the molecules ID's and their intensity profile.

Each chromosome was then analyzed independently using an automation script *exexute_tasks* that supports a **YML** file format for each script's specific arguments.

Using the script *CheckProfileAlignmentA.py* we extracted the data from the **profileTrace.txt** file and combined it with the alignment data (Xmap format) to account for the intensity of the red EcoDam labelling channel at each genomic location. The output of this script is the intensity of the red channel for every molecule at each genomic position to which the molecule was aligned, in bp resolution. The *Convert_output_file.py* script converted the data to a two column CSV file, representing the genomic location and the **average** intensity value from all DNA molecules mapped to that location.

Then *csv_means_to_BEDgraphA.py* script was used to alter the data to the visual representation format, bedgraph (BED format for visualization in standard genome browsers such as the UCSC Genome Browser (https://genome.ucsc.edu/index.html).[6] While also taking into account the resolution limitation of OGM, this script expands the locations of the reported signals (reported at bp resolution) to 500 bp. Defining 250 bp upstream as the start position and 250 bp downstream as the end position.

After applying the whole pipeline on both chromatin and naked samples they were each merged to a Whole genome file containing all 23 female chromosomes for GM12878. For the final step, the naked data was used for the chromatin normalization.

4. **Data smoothing**

Data was smoothed using a Gaussian sliding window. The window size and STD were chosen to yield the best correlation with the expected theoretical profile predicted by the genomic DAM site distribution. We screened different combinations of STD and window size for smoothing, and calculated the correlation coefficient (CC) between the smoothed theoretical data and the naked control experimental data. This was done in an iterative manner, so that each time one parameter was fixed, and the other was monitored, until finding the pair with the best CC value (fig S1 A&B). To ease computation resources CC was not calculated over the whole genome but rather on a representative region (chr19: 132000-155000). The optimal parameters were found to be a window size of 4000bp and STD value of 2000bp. Indeed, applying these values over the whole genome generated theoretical peaks that highly resemble our experimental peaks (fig S1C).

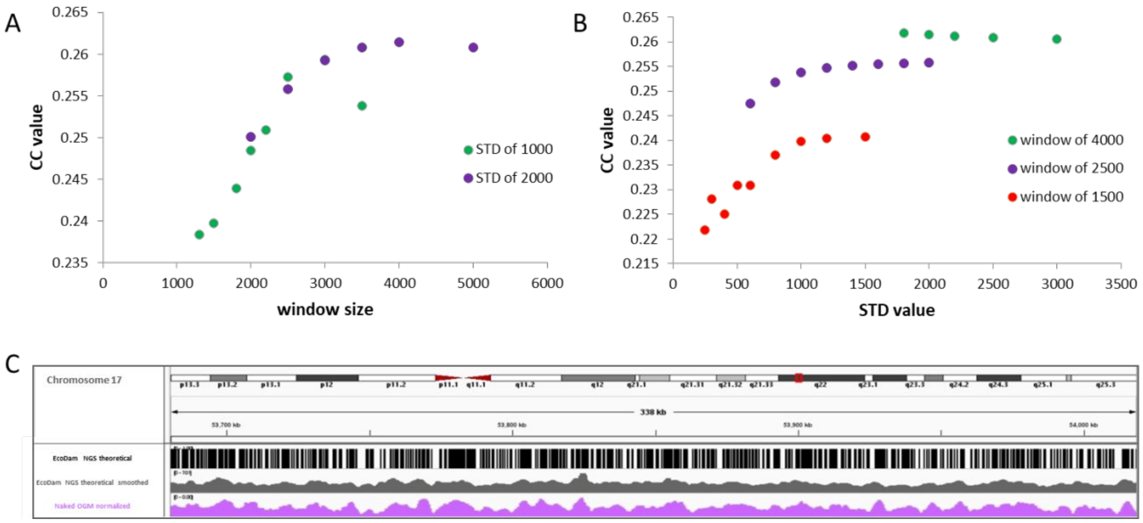

Figure S1. A. CC values for different window sizes while the STD value is fixed either on 1000 or 2000 bp. B. CC values for different STD values while window sizes is fixed on 4000, 2500 or 1500 bp. C. Genome browser screenshot, top to bottom; EcoDam NGS Theoretical track, smoothed EcoDam theoretical track (window size 4000 bp and STD 2000 bp), naked OGM normalized track.

5. **Correlation between DAFCA replicates**

For assay reproducibility assessment, we performed the experiment on two biological replicates of the GM12878 cell line and calculated their Pearson correlation coefficient across chromosome 10, we divided the genome into non-overlapping windows of 500bp, 5kbp and 50kpb and calculated the average DAFCA signal separately for each window in both replicates (BEDTools map). For each of the window sizes we calculated the Pearson correlation between average signals of both replicates, the resulting values were 0.63, 0.77 & 0.85 for the 500bp, 5kb and 50kb bins

respectively. Scatter plots demonstrating the correlation are shown in figure S2. As expected in correlated samples the dots display a diagonal behavior. This diagonal is shifted from the origin due to shifts in the base-line fluorescent level between experiments. This shift is corrected for data further down the analysis during the normalization step

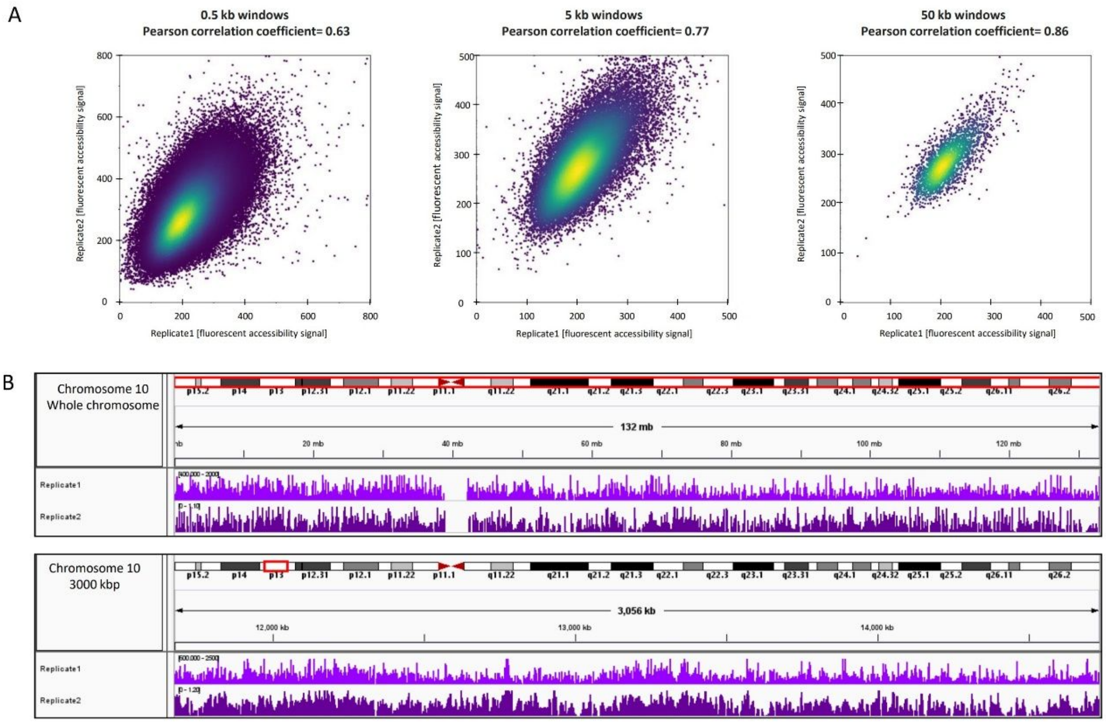

Figure S2. A. Scatter plots demonstrating the correlation between replicates. Each dot represents the average DAFCA signal in windows along the genome for each replicate. Three scatter plots are shown displaying values for windows of 0.5 kbp, 5 kbp and 50 kbp. B. Genome browser screenshot comparing the two replicates at whole chromosome view and 3 Mbp.

6. References

(1) Frankish, A.; Diekhans, M.; Ferreira, A. M.; Johnson, R.; Jungreis, I.; Loveland, J.; Mudge, J. M.; Sisu, C.; Wright, J.; Armstrong, J.; Barnes, I.; Berry, A.; Bignell, A.; Carbonell Sala, S.; Chrast, J.; Cunningham, F.; Di Domenico, T.; Donaldson, S.; Fiddes, I. T.; García Girón, C.; *et al*. GENCODE Reference Annotation for the Human and Mouse Genomes. *Nucleic Acids Res.* **2019**, *47*, 766–773. doi: v10.1093/nar/gky955.

(2) Cao, Q.; Anyansi, C.; Hu, X.; Xu, L.; Xiong, L.; Tang, W.; Mok, M. T. S.; Cheng, C.; Fan, X.; Gerstein, M.; Cheng, A. S. L.; Yip, K. Y. Reconstruction of Enhancer-Target Networks in 935 Samples of Human Primary Cells, Tissues and Cell Lines. *Nat. Genet.* **2017**, *49*, 1428–1436. doi: 10.1038/ng.3950.

(3) Haeussler, M.; Zweig, A. S.; Tyner, C.; Speir, M. L.; Rosenbloom, K. R.; Raney, B. J.; Lee, C. M.;

Lee, B. T.; Hinrichs, A. S.; Gonzalez, J. N.; Gibson, D.; Diekhans, M.; Clawson, H.; Casper, J.; Barber, G. P.; Haussler, D.; Kuhn, R. M.; Kent, W. J. The UCSC Genome Browser Database: 2019 Update. *Nucleic Acids Res.* **2019**, *47* , 853–858. doi: 10.1093/nar/gky1095.

(4) Amemiya, H. M.; Kundaje, A.; Boyle, A. P. The ENCODE Blacklist: Identification of Problematic Regions of the Genome. *Sci. Rep.* **2019**, *9*, 1–5. doi: 10.1038/s41598-019-45839-z.

(5) Quaas, R.; Georgalis, Y.; Saenger, W.; Hahn, U. High-Level Expression of a Semisynthetic Dam Gene in Escherichia. **1991**, *98*, 83–88.

(6) Kent, W. J.; Sugnet, C. W.; Furey, T. S.; Roskin, K. M.; Pringle, T. H.; Zahler, A. M.; Haussler, D. The Human Genome Browser at UCSC. **2002**, 996–1006. doi: 10.1101/gr.229102.
